# A Systemic Inflammatory Endotype of Asthma With More Severe Disease Identified by Unbiased Clustering of the Serum Cytokine Profile

**DOI:** 10.1097/MD.0000000000003774

**Published:** 2016-06-24

**Authors:** Zhenyu Liang, Laiyu Liu, Haijin Zhao, Yang Xia, Weizhen Zhang, Yanmei Ye, Mei Jiang, Shaoxi Cai

**Affiliations:** From the Department of Respiratory and Critical Care Medicine, Chronic Airways Diseases Laboratory, Nanfang Hospital, Southern Medical University (ZL, LL, HZ, YX, WZ, YY, SC); and State Key Laboratory of Respiratory Disease, National Clinical Research Center for Respiratory Disease, Guangzhou Institute of Respiratory Disease, First Affiliated Hospital of Guangzhou Medical University, Guangzhou, China (ZL, MJ).

## Abstract

Asthma is considered as a clinical and molecularly heterogeneous disorder. Systemic inflammation is suggested to play an important role in a group of asthma patients. We hypothesized that there is a subgroup of patients with asthma characterized by systemic inflammation. In this study, we aimed to discriminate asthma subtypes based on circulating biomarkers and to determine whether a systemic inflammatory endotype of asthma could be identified. In the present cross-sectional study, 50 patients with untreated asthma were prospectively recruited from a single academic outpatient clinic, and characterized with respect to clinical, functional, and inflammatory parameters. The expression profiles of 20 serum cytokines were assessed by anti-human cytokine antibody array. Then, hierarchical clustering analysis was performed based on principal component analysis (PCA)-transformed data to classify the clinical groups. PCA showed that 6 independent components accounted for 80.113% of the variance, and PCA-based hierarchical clustering identified 3 endotypes. One of the endotypes was evidenced by elevated systemic inflammation markers such as leptin, vascular endothelial growth factor (VEGF), and reduced levels of soluble receptor for advanced glycation end products (sRAGE), an anti-inflammatory molecule. More female patients were included, with higher circulating neutrophil counts and more severe symptoms. In conclusion, we identified an endotype of asthma characterized by systemic inflammation and severe symptoms. Increased levels of VEGF, leptin and decreased level of sRAGE may contribute to the systemic inflammation of this asthma endotype.

## Abstract 2

Supplemental Digital Content is available in the text

## INTRODUCTION

Asthma is a heterogeneous condition with complex underlying mechanisms.^1^ Asthma endotypes are defined based on distinct pathophysiological mechanisms, therefore reflecting the corresponding mechanisms.^1–3^ Analysis of endotypes might help better understand asthma mechanisms. Recently, the role of systemic inflammation in patients with asthma has attracted increasing attention. For instance, Wood et al showed that augmented systemic inflammation (elevated IL-6 and high-sensitivity C-reactive protein levels) characterized a group of asthmatic patients with neutrophilic airway inflammation, and was associated with worse clinical outcomes.^4^ In addition, a concomitant deficiency of soluble receptor for advanced glycation end products (sRAGE) was observed in neutrophilic asthma.^4,5^

Therefore, we inferred that systemic inflammation might play an important role in a group of asthma patients, thus representing an endotypic characteristic of asthma. We hypothesized that there is an asthma endotype with relatively high grade of systemic inflammation. To test our hypothesis, we assessed the profiles of circulating cytokines in patients with well-characterized asthma using cytokine microarray analyses, and performed unbiased/unsupervised cluster analysis on the profiles data. The cytokines studied included common markers of systemic inflammation (interleukin [IL]-6, tumor necrosis factor [TNF]-α, IL-8, and leptin), a Th1-specific cytokine (interferon [INF]-γ), Th2-related cytokines (IL-4, IL-5, IL-13, granulocyte-macrophage colony-stimulating factor [GM-CSF], thymic stromal lymphopoietin [TSLP], and IL-33), Th17/Treg cytokines (IL-17, IL-23, and IL-10), growth factors (vascular endothelial growth factor [VEGF], epidermal growth factor [EGF], and transforming growth factor [TGF]-β1), anti-inflammatory (sRAGE), and others (IL-9 and IL-1β). To take into consideration the redundancy of multiple variables, principal component analysis (PCA) was performed before clustering analysis, and clinical systemic inflammatory characteristics were compared among clusters.

## PATIENTS AND METHODS

### Patients

In the present prospective cross-sectional study, 50 untreated asthmatics in the nonacute episode phase were recruited at the Department of Respiratory and Critical Care Medicine, Nanfang Hospital, Southern Medical University (Guangzhou, China) between July 2012 and July 2013. Inclusion criteria were: age >18 years; initially diagnosed in our facility according to the Global Initiative for Asthma (GINA) guidelines^6^; positive bronchodilator reversibility test (>12% and 200-mL increase in forced expiratory volume in one second (FEV_1_) after a 400-μg salbutamol inhalation) or methacholine provocation test; and steroid-naïve. Exclusion criteria were: respiratory tract infection based on chest x-ray (every patient underwent chest x-ray) within the past 4 weeks; any airway disease other than asthma; peripheral white blood cell (WBC) count outside the normal range; or currently smoking. Informed consent was obtained from all patients. The study was approved by the ethics committee of Southern Medical University (approval No.: 2012–072).

Data collected at enrollment included patient demographic characteristics, pulmonary function data, 5-item asthma control questionnaire (ACQ-5),^7^ and symptom score (daytime and nighttime)^8–10^ of asthmatics before induction of sputum, which was collected for cell differential count. Venous blood samples were collected from all subjects and separated at the same visit. Serum total IgE concentrations and cytokine profiles were determined using electrochemiluminescence and customized Quantibody array, respectively.

### Pulmonary Function Tests

Spirometry was performed before sputum induction using the Jaeger Masterscope spirometry system (Jaeger, Wuerzburg, Germany) according to the American Thoracic Society (ATS) guidelines.^11^

### Blood Samples, Sputum Induction, and Processing

Venous blood samples were collected in ethylenediamine tetraacetic acid (EDTA) anticoagulation tubes before sputum induction. Then, differential white blood cell count was carried out on a Coulter instrument (Sysmex-XE2100, Kobe, Japan).

Sputum induction and processing were performed following the guidelines suggested by the Task Force of the European Respiratory Society.^12,13^

### Microarray Analysis of Serum Cytokine Profiles

The levels of INF-γ, IL-4, IL-5, IL-13, GM-CSF, TSLP, IL-33, IL-17, IL-23, IL-10, IL-6, TNF-α, IL-8, leptin, VEGF, EGF, TGF-β1, IL-9, IL-1β, and sRAGE in serum samples were determined in duplicate with a customized microarray (Human Cytokine Antibody Microarray slides; RayBiotech, Inc. Norcross, GA, USA).

### Statistical Analysis

Data are expressed as mean ± SD for continuous variables, and comparisons among groups were performed by 1-way analysis of variance (ANOVA) with the least significant difference (LSD) post hoc test. Variables with skewed distribution were expressed as median [interquartile range (P25–P75)], and comparisons among groups were carried out using the Kruskal-Wallis test with the Nemenyi post hoc test. For categorical variables, the number of observations and percentages were given in each category. All statistical analyses were performed with the SPSS software (version 19.0; SPSS Inc, Chicago, IL).

Unbiased/unsupervised agglomerative (“bottoms-up”) hierarchical clustering was performed on *Z* standardized data by using the uncentered correlation as the similarity metric (EisenLab Cluster version 2.11; Eisen Lab, Stanford, CA, USA). The dendrogram and resulting heatmap were visualized using TreeView (version 1.60; Eisen Lab, Stanford, CA, USA). PCA was performed on these variables, and hierarchical clustering was carried out on principal components of the PCA.

## RESULTS

### Hierarchical Clustering Without PCA Pretreatment

The clinical characteristics of the 50 asthma subjects are shown in Table 1. Mean age was 39.7 ± 12.4 years and the sex ratio was nearly 1. Most patients had a normal body mass index (BMI), with a mean of 21.95 ± 3.25. Atopy was present in 27 (54%) patients.

**TABLE 1:**
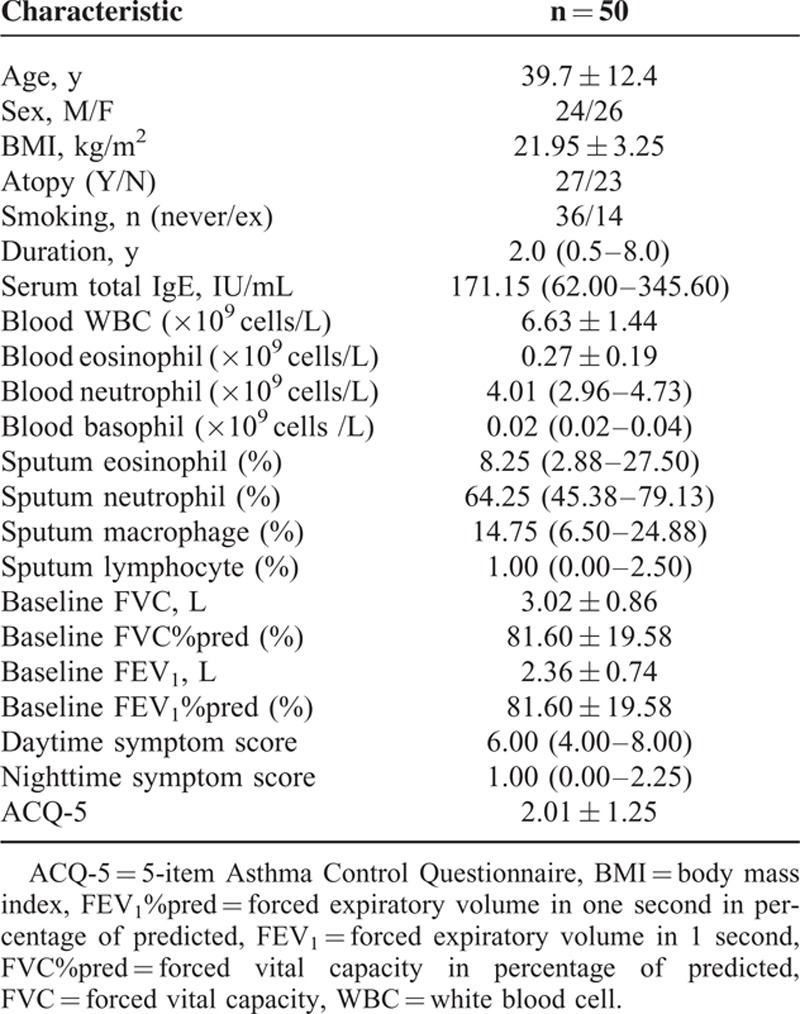
Demographics and Clinical Characteristics of all Subjects

To confirm the PCA yield as a preliminary step before hierarchical clustering, we performed hierarchical clustering analysis based on the total number of initial variables without PCA pretreatment. Using the hierarchical clustering approach, a dendrogram and a heat map were generated (Figure S1). Based on Figure S1, 3 clusters were identified, and the cytokine levels and clinical features in the 3 groups were compared with one another. As shown in Table S1, 14 (EGF, GM-CSF, IFN-γ, IL-4, IL-5, IL-6, IL-8, IL-9, IL-10, IL-13, IL-17, IL-23, TGF-β1, and TNF-α) of 20 cytokines were statistically different among these groups (all *P* < 0.05), whereas most demographic and clinical parameters (age, gender, BMI, family history, atopy, smoking history, and biochemistry) were similar among the groups (Table S2). These findings suggested that the subtypes identified by clustering without the initial PCA might be less clinically relevant because of the lack of differences among the groups in clinical characteristics.

### PCA

**FIGURE 1:**
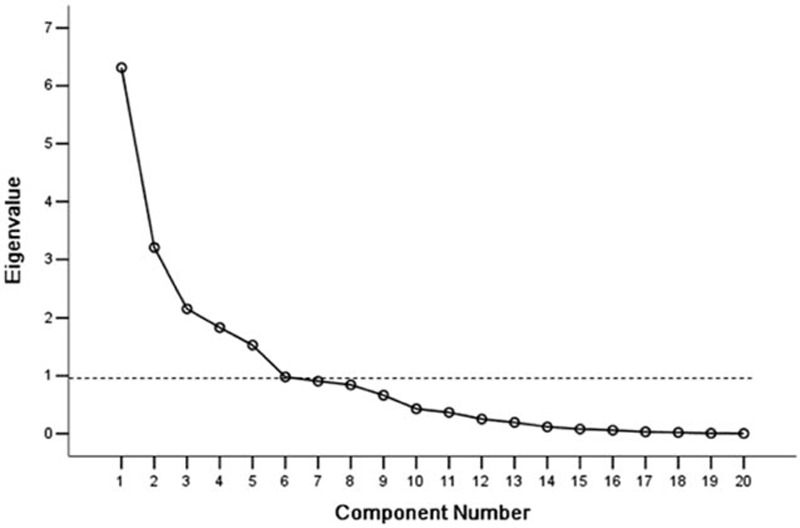
Screeplot of principal component analysis. Six components had eigen values ≥1 (dotted line, and because 0.981 is approximately equal to 1, we also captured the sixth component)) and explained 80.113% of the variance.

Cytokine profile data were processed with PCA, and the 6 largest principal components extracted explained 80.113% of the information contained in the original data (Figure 1, Tables S3 and S4), suggesting that these 6 components alone explained most of the variability among groups.

### Hierarchical Clustering Based on PCA

**FIGURE 2:**
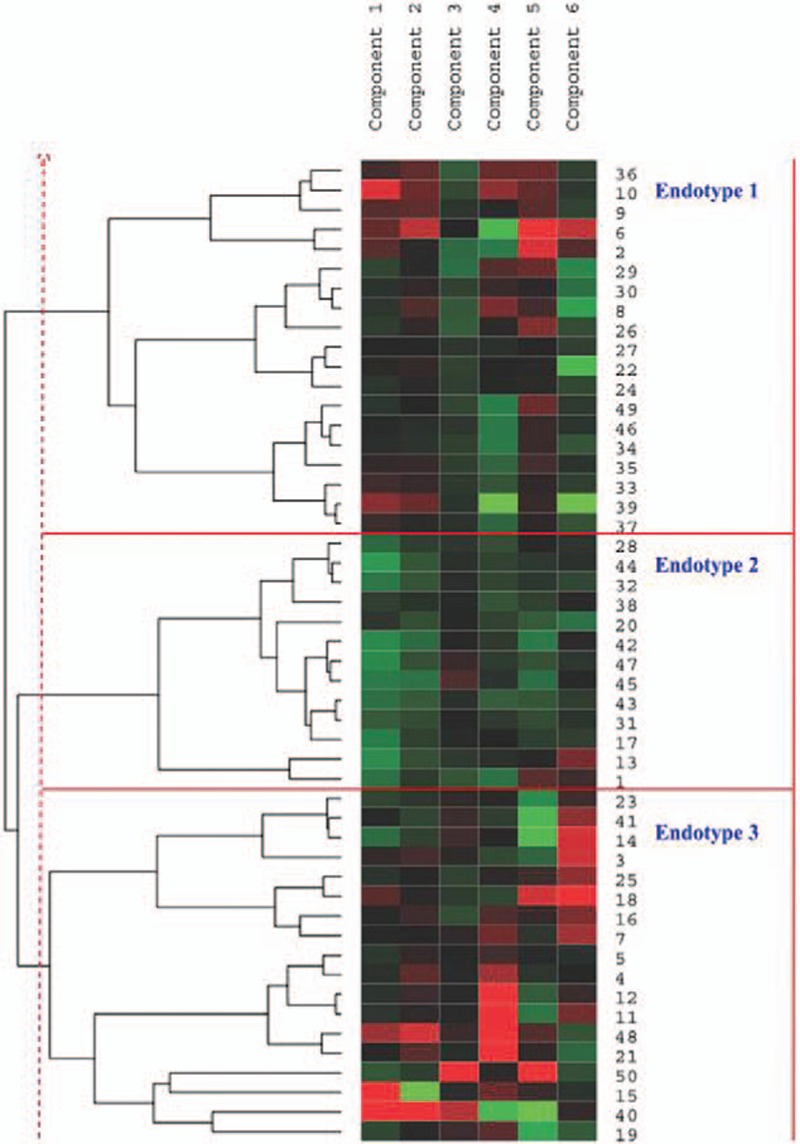
Hierarchical clustering based on PCA. Each column is a component, and each row is an individual patient. Numbers at the right side of the heat map are the patient numbers. Left, dendrogram showing similarity of groups. Right, 3 endotypes are indicated by vertical bars.

Using PCA, 3 endotypes of asthmatics with distinct molecular characteristics were identified based on the 6 principal components obtained using hierarchical clustering analysis (Figure 2).

### Molecular Characteristics of the 3 Endotypes

**FIGURE 3:**
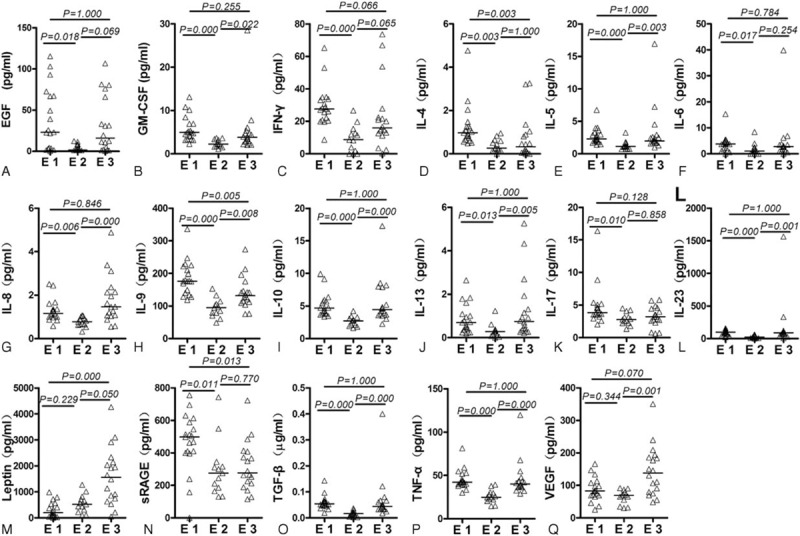
Pairwise comparisons of serum cytokine concentrations between endotypes. Data were analyzed with 1-way analysis of variance with the least significant difference post hoc test. E = endotype, EGF = epidermal growth factor, GM-CSF = Granulocyte-macroprhage colony-stimulating factor, IFN = interferon, IL = interleukin, sRAGE = soluble receptor for advanced glycation end products, TGF-β1 = transforming growth factor-beta 1, TNF = tumor necrosis factor, VEGF = vascular endothelial growth factor.

Post-hoc analyses of between-group differences were performed. Compared with endotypes 2 and 3, endotype 1 showed relatively high levels of proinflammatory cytokines (IFN-γ, IL-4, IL-5, IL-6, IL-9, IL-17, IL-23, EGF, GM-CSF, and TNF-α) and relatively high levels of anti-inflammatory cytokines (IL-10, TGF-β, and sRAGE). Compared with endotypes 1 and 3, endotype 2 showed relatively low levels of proinflammatory cytokines (INF-γ, IL-4, IL-5, IL-6, IL-8, IL-9, IL-13, IL-17, IL-23, EGF, GM-CSF, TNF-α, and VEGF), and relatively low levels of anti-inflammatory cytokines (IL-10 and sRAGE). Compared with endotypes 1 and 2, endotype 3 displayed relatively high levels of leptin and VFGF, but low sRAGE levels (Table 2 and Figure 3). These results suggest distinct patterns of cytokines among there 3 endotypes.

**TABLE 2:**
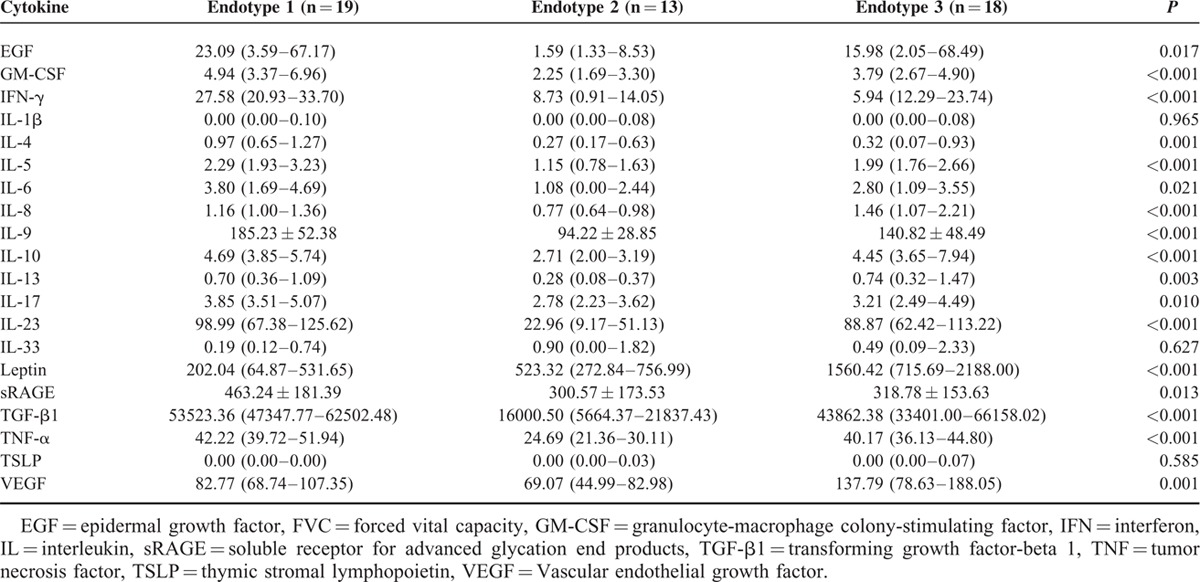
Comparison of the Serum Cytokine Concentrations Among the 3 Endotypes Identified by the PCA-based Hierarchical Clustering (pg/mL)

### Clinical Characteristics of the 3 Endotypes

**FIGURE 4:**
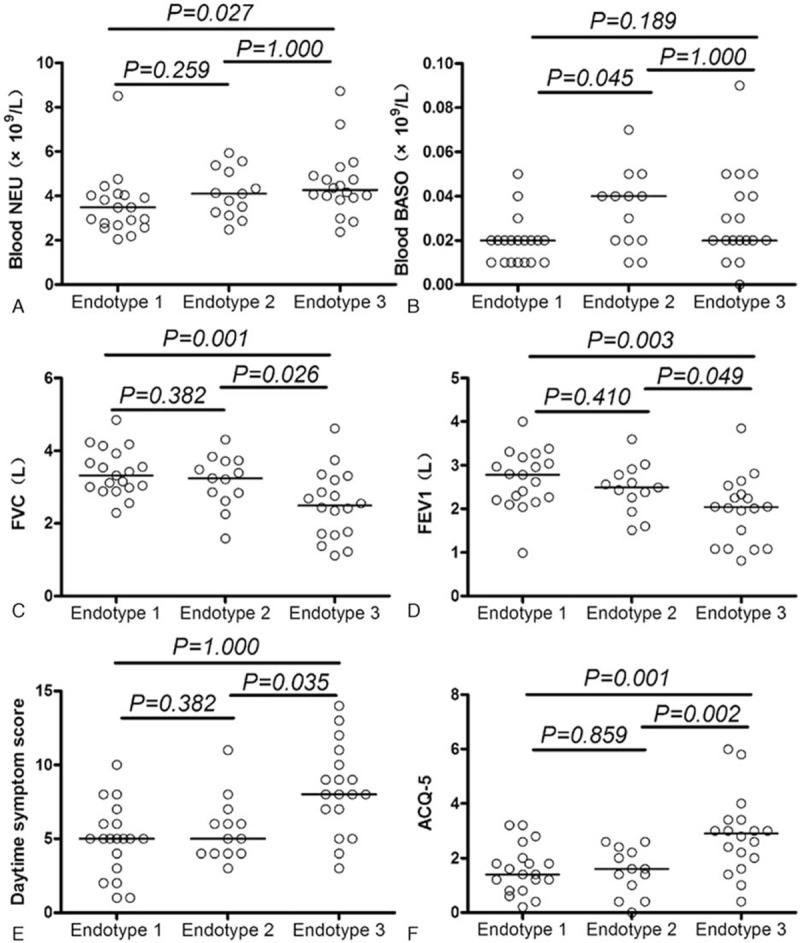
Pairwise comparisons of clinical parameters between endotypes. Data were analyzed with one-way analysis of variance with the least significant difference post hoc test. ACQ-5 = 5-item Asthma Control Questionnaire, FEV1 = forced expiratory volume in 1 second, FVC = forced vital capacity

To determine whether the patients within these endotypes represented clinically distinct subgroups of asthma, the clinical features of the 3 endotypes were analyzed (Table 3 and Figure 4). Endotype 1 showed a higher proportion of males, low blood basophil levels, high baseline forced vital capacity (FVC), high baseline FEV1, and low ACQ-5. Endotype 2 showed high blood neutrophil levels, high blood basophil levels, high FVC, high FEV1, and low ACQ-5. Finally, endotype 3 showed a high proportion of females, high blood neutrophil levels, low blood basophil levels, low baseline FVC, low baseline FEV1, high datime symptom score, and high ACQ-5 score. Therefore, both endotypes 1 and 2 had a higher frequency of patients with relatively normal lung function and moderate symptoms, although endotype 1 contained significantly more male patients. Endotype 3 had a higher-frequency female patients, and was characterized by decreased lung function and more severe symptoms (Figure 4).

**TABLE 3:**
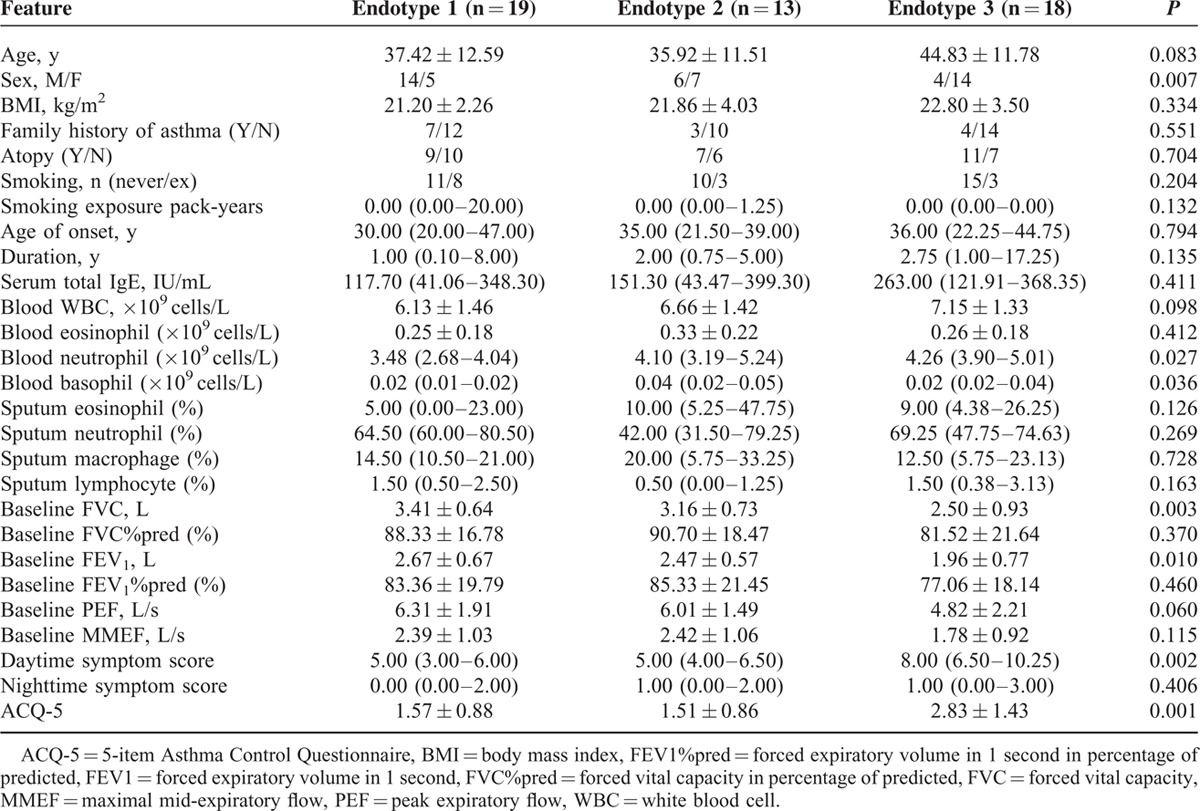
Comparison of Demographic and Clinical Characteristics among the 3 Endotypes Identified by the PCA-based Hierarchical Clustering

## DISCUSSION

In the present exploratory study, 20 biological variables were quantitatively analyzed for studying asthma endotypes using clustering analysis based on PCA, which has been seldom used previously to assess asthma subtypes in patients.^14^ We provide herein the first preliminary evidence for circulating biological variables for identifying asthma subtypes. Despite the small sample size, the results are very promising. As shown above, 3 distinct clinical groups of asthma patients were identified. The endotype 1, high proinflammatory and anti-inflammatory subtype, was enriched in male patients with relatively normal lung function and moderate symptoms. The lower proinflammatory and anti-inflammatory (IL-10 and sRAGE) subtype, endotype 2, was also enriched in patients with relatively normal lung function and moderate symptoms. The systemic inflammatory subtype, endotype 3, exhibited the characteristics of systemic inflammation, as evidenced by increased systemic inflammation markers such as leptin, VEGF, and circulating neutrophils concomitant with decreased sRAGE levels; endotype 3 patients were mainly female with lower lung function and more severe symptoms, but the lower lung function might be because of the higher proportion of females, which will need further exploration. Therefore, as underlined by Haldar et al,^14^ PCA offers new opportunities to achieve a better characterization of asthma, which might lead to new treatment approaches. However, differences in patient populations and variables make the direct comparison difficult between the 2 studies, and additional studies are necessary.

To reduce the redundancy of asthmatic serum microarray data, PCA was used before clustering analysis, unlike many clustering analyses of asthma phenotypes. Indeed, the present study suggested that subtypes identified by clustering without initial PCA may be less clinically relevant, and data redundancy should be taken into consideration.^15,16^

As predicted, a special subtype (endotype 3) of more severe asthma was found, in which systemic inflammation might play a role. In 2012, Wood et al^4^ reported an association between neutrophilic asthma and systemic inflammation. In fact, multiple chronic noncommunicable diseases (CNCDs) such as type 2 diabetes mellitus, cardiovascular diseases, and cancer have been reported to be associated with chronic low-grade systemic inflammation.^17^ Moreover, leptin has been demonstrated to be involved in systemic inflammation.^18,19^ As shown above, endotype 3 displayed high levels of serum leptin and circulating neutrophil counts, and was enriched in female patients with low lung function and relatively severe symptoms. In addition, a positive correlation was observed between serum leptin levels and blood neutrophil counts (*r* = 0.312, *P* = 0.028; female: *r* = 0.459, *P* = 0.018). In line with previous findings,^20^ these data indicated that leptin may be involved in the systemic inflammation and severity of asthma. However, endotype 3 subjects did not show higher BMI, in agreement with previous findings indicating that leptin may be critically involved in the pathogenesis of asthma^21^; therefore, endotype 3 represented a systemic inflammatory subtype, independent of obesity. However, all subjects in this study were Asians, which have been reported to have lower BMI but higher percent body fat, compared with whites.^22^

Notably, serum VEGF was increased in endotype 3. Park et al^23^ reported that serum VEGF is associated with the severity of systemic inflammation in patients with inflammatory lung disease. In addition, leptin increased the gene expression and protein level of VEGF in human hepatic stellate cells.^24^ In concert, our data bring strong evidence suggesting that VEGF may also contribute to systemic inflammation and asthma severity.

As shown above, both endotypes 1 and 3 showed high levels of proinflammatory cytokines. However, endotype 1 also displayed high sRAGE levels, whereas endotype 3 had relatively low levels. The receptor for advanced glycation end-products (RAGE) is a pattern-recognition receptor accounting for the host response to injury, infection, and inflammation. Indeed, the ligand-RAGE pathway has been recognized as a key pathway in a wide range of chronic diseases. RAGE is a membrane receptor, but also has soluble forms (sRAGE), which can function as decoy receptor of RAGE,^25^ competitively binding to damage-associated molecular patterns such as HMGB1 and HSP70 and blocking the induced inflammation.^10,25,26^ Deficiencies in sRAGE are associated with increased inflammation in various chronic conditions, including chronic obstructive pulmonary disease (COPD).^5,27^ As deficiencies in sRAGE are linked to neutrophilic asthma, COPD, and other chronic inflammatory diseases,^5,27^ our results suggested that sRAGE deficiency may be the reason behind the development of systemic inflammation in a special group of asthma patients.

Several limitations of the current exploratory study should be mentioned. First, this was a cross-sectional study, and it is possible for asthma treatments to have disease-modifying effects that affected the molecular characteristics of disease subtypes. Second, this study was performed on subjects with untreated asthma, and longitudinal follow-up study is needed to improve our knowledge of treatment response and natural history of subjects within these endotypes. Third, because of the small sample size, it is possible that more endotypes were not identified. Fourth, inclusion of other biological markers, for example, exhaled nitric oxide fraction (FeNO) and circulating C-reaction protein, may increase our knowledge of the clinical–biological characteristics of the endotypes.

## CONCLUSION

Overall, unbiased analysis of serum cytokine profiles contributed to identifying a clinically and biologically distinct subtype of asthma. Despite the small sample size, the results are very promising. A more severe asthma endotype with systemic inflammation was identified, with increased leptin, VEGF, circulating neutrophil levels, and decreased level of sRAGE, an anti-inflammatory molecule; the patients of this endotype suffered from rather poor lung function and more severe symptoms. These results provide the first evidence suggesting that analysis of serum cytokine profiles is useful for asthma endotyping. The systemic inflammatory endotype of asthma identified in this study represents a specific subtype with different underlying pathophysiology compared with milder subtypes. Future studies should include larger sample size and prospective follow-up studies with a focus on treatment response and natural history, to eventually design targeted treatment or personalized therapy for asthma.

## Supplementary Material

Supplemental Digital Content
