# Supplementary material for: A Systemic Inflammatory Endotype of Asthma With More Severe Disease Identified by Unbiased Clustering of the Serum Cytokine Profile
Source: Medicine (Baltimore). 2016 Jun 24;95(25):e3774. doi: 10.1097/MD.0000000000003774 (PMC4998303; doi:10.1097/MD.0000000000003774)
Supplement: Supplemental Digital Content [file medi-95-e3774-s001.doc]

**
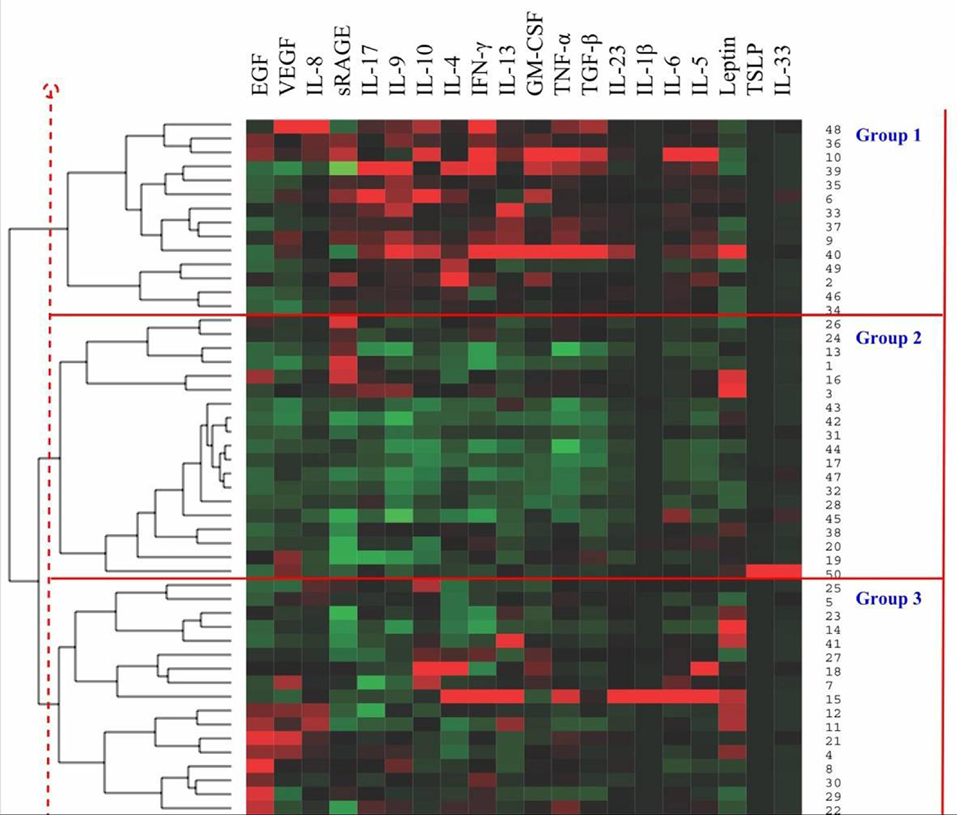
**

**FIGURE S1.** Original hierarchical cluster analysis of 20 cytokines

The cluster tree and heat map were performed on the z-score normalized cytokine data. Each column is a cytokine, and each row is an individual patient. Numbers at the right side of the heat map are the patient numbers. Left dendrogram showed similarity of groups. Right, three groups are indicated by vertical bars.

**TABLE S1.** Comparison of the serum cytokine concentrations among the three groups identified by the original hierarchical clustering (pg/ml)

| Cytokine | Group 1 (n=14) | Group 2 (n= 19) | Group 3 (n=17) | *P* value |
| --- | --- | --- | --- | --- |
| EGF | 4.07 (1.58-29.23) | 7.46 (1.41-12.39) | 33.30 (8.01-87.25) | 0.017 |
| GM-CSF | 5.22 (3.98-10.30) | 2.84 (1.77-3.64) | 3.65 (2.35-4.86) | <0.001 |
| IFN-γ | 29.54 (20.31-53.16) | 12.65 (3.95-21.71) | 16.69 (12.15-27.34) | 0.002 |
| IL-1β | 0.03 (0.00-0.14) | 0.00 (0.00-0.08) | 0.00 (0.00-0.09) | 0.696 |
| IL-4 | 1.06 (0.90-1.61) | 0.45 (0.19-0.65) | 0.46 (0.06-1.04) | <0.001 |
| IL-5 | 2.96 (2.27-3.98) | 1.49 (1.04-1.82) | 1.97 (1.65-2.47) | <0.001 |
| IL-6 | 4.43 (2.95-5.38) | 1.26 (0.00-2.41) | 3.00 (1.04-3.74) | 0.001 |
| IL-8 | 1.32 (0.99-1.87) | 0.82 (0.62-1.07) | 1.28 (1.05-2.14) | <0.001 |
| IL-9 | 210.20±51.67 | 106.52±38.52 | 136.02±29.04 | <0.001 |
| IL-10 | 5.59 (4.60-8.07) | 2.86 (2.12-3.68) | 4.10 (3.68-5.79) | <0.001 |
| IL-13 | 1.05 (0.71-1.80) | 0.30 (0.16-0.41) | 0.57 (0.28-1.12) | <0.001 |
| IL-17 | 4.59 (3.80-5.47) | 2.89 (2.24-4.08) | 2.94 (2.42-3.74) | <0.001 |
| IL-23 | 116.36 (92.58-132.60) | 37.40 (9.74-62.48) | 89.76 (58.75-114.39) | <0.001 |
| IL-33 | 0.28 (0.12-1.46) | 0.74 (0.08-1.23) | 0.15 (0.06-0.98) | 0.573 |
| Leptin | 225.09 (85.81-735.24) | 568.05 (325.32-1011.86) | 744.94 (259.67-1936.74) | 0.134 |
| sRAGE | 451.78±187.83 | 358.16±211.72 | 312.77±115.21 | 0.100 |
| TGF-β1 | 62227.6 (52356.9-10215.8) | 21225.0 (15001.3-47347.8) | 41523.3 (32751.2-47888.2) | <0.001 |
| TNF-α | 44.03 (40.16-55.79) | 29.55 (22.59-41.22) | 38.86 (34.30-43.49) | <0.001 |
| TSLP | 0.00 (0.00-0.18) | 0.00 (0.00-0.02) | 0.00 (0.00-0.01) | 0.080 |
| VEGF | 81.73 (67.69-147.87) | 72.73 (65.39-90.54) | 97.65 (72.10-164.95) | 0.142 |

EGF, epidermal growth factor; sRAGE: Soluble receptor for advanced glycation end products; FVC: Forced vital capacity; GM-CSF: Granulocyte-macrophage colony-stimulating factor; TGF-β1: transforming growth factor-beta 1; TSLP: Thymic stromal lymphopoietin; VEGF: Vascular endothelial growth factor.

**TABLE S2. Comparison of demographic and clinical characteristics among the three groups identified by the original hierarchical clustering**

| Feature | Group 1(n=14) | Group 2(n=19) | Group 3(n=17) | *P* value |
| --- | --- | --- | --- | --- |
| Age, yrs | 38.79±13.31 | 38.32±12.54 | 42.00±11.88 | 0.647 |
| Gender, M/F | 9/5 | 8/11 | 7/10 | 0.355 |
| BMI (kg/m²) | 21.81±3.24 | 21.60±3.73 | 22.45±2.78 | 0.729 |
| Family history of asthma (Y/N) | 7/7 | 3/16 | 4/13 | 0.085 |
| Atopy (Y/N) | 8/6 | 10/9 | 9/8 | 0.962 |
| Smoking, n (Never/Ex) | 5/9 | 5/14 | 4/13 | 0.738 |
| Smoking exposure pack-yrs | 0.00 (0.00-11.25) | 0.00 (0.00-2.5) | 0.00 (0.00-0.50) | 0.557 |
| Age of onset, yrs | 40.00 (20-47.25) | 35.00 (21.00-43.00) | 35.00 (27.00-40.50) | 0.922 |
| Duration, yrs | 1.00 (0.10-7.50) | 2.00 (1.00-6.00) | 2.50 (1.00-12.50) | 0.314 |
| Serum total IgE (IU/ml) | 142.30 (47.74-428.18) | 158.80 (58.37-440.80) | 246.30 (92.80-334.70) | 0.807 |
| Blood WBC (×10^9^/L) | 6.01±0.96 | 6.74±1.50 | 7.03±1.61 | 0.135 |
| Blood eosinophil (×10^9^/L) | 0.27±0.19 | 0.29±0.21 | 0.26±0.18 | 0.919 |
| Blood neutrophil (×10^9^/L) | 3.66 (2.80-4.06) | 4.10 (3.12-5.38) | 4.06 (2.88-4.75) | 0.162 |
| Blood basophil (×10^9^/L) | 0.02 (0.01-0.03) | 0.02 (0.02-0.04) | 0.02 (0.02-0.05) | 0.386 |
| Sputum eosinophil (%) | 5.63 (1.88-25.25) | 9.00 (5.50-37.00) | 9.00 (2.50-24.50) | 0.413 |
| Sputum neutrophil (%) | 74.25 (58.63-80.69) | 54.75 (36.00-79.00) | 66.50 (55.50-74.00) | 0.140 |
| Sputum macrophage (%) | 14.75 (10.00-19.88) | 20.00 (6.50-30.50) | 10.50 (5.50-23.00) | 0.443 |
| Sputum lymphocyte (%) | 0.50 (0.00-2.00) | 1.00 (0.00-1.50) | 2.00 (1.00-3.25) | 0.025 |
| Baseline FVC (L) | 3.14±0.67 | 3.06±0.70 | 2.88±1.15 | 0.695 |
| Baseline FVC%pred (%) | 87.19±20.93 | 90.38±16.33 | 81.58±20.41 | 0.389 |
| Baseline FEV1 (L) | 2.37±0.71 | 2.39±0.58 | 2.33±0.94 | 0.967 |
| Baseline FEV1%pred (%) | 77.98±22.62 | 85.06±19.17 | 80.73±17.83 | 0.585 |
| Daytime symptom score | 5.50 (3.50-8.00) | 6.00 (4.00-7.00) | 6.00 (4.50-9.50) | 0.774 |
| Nighttime symptom score | 0.50 (0.00-2.0) | 0.00 (0.00-1.00) | 1.00 (0.00-3.00) | 0.306 |
| ACQ-5 | 2.14±1.42 | 1.65±0.87 | 2.31±1.44 | 0.272 |

BMI: body mass index, WBC: white blood cell, FEV1: forced expiratory volume in 1 second, FVC: forced vital capacity, FEV1%pred: forced expiratory volume in one second in percentage of predicted, FVC%pred: forced vital capacity in percentage of predicted, ACQ-5: 5-item Asthma Control Questionnaire

**TABLE S3.** Total Variance Explained

| Component | Initial Eigenvalues | | |  | Extraction Sums of Squared Loadings | | |
| --- | --- | --- | --- | --- | --- | --- | --- |
|  | Eigenvalues | Variance (%) | Cumulative (%) |  | Eigenvalues | Variance (%) | Cumulative (%) |
| 1 | 6.314 | 31.570 | 31.570 |  | 6.314 | 31.570 | 31.570 |
| 2 | 3.206 | 16.029 | 47.599 |  | 3.206 | 16.029 | 47.599 |
| 3 | 2.157 | 10.787 | 58.386 |  | 2.157 | 10.787 | 58.386 |
| 4 | 1.833 | 9.166 | 67.552 |  | 1.833 | 9.166 | 67.552 |
| 5 | 1.531 | 7.657 | 75.209 |  | 1.531 | 7.657 | 75.209 |
| 6 | 0.981 | 4.904 | 80.113 |  | 0.981 | 4.904 | 80.113 |
| 7 | 0.907 | 4.536 | 84.649 |  |  |  |  |
| 8 | 0.844 | 4.220 | 88.869 |  |  |  |  |
| 9 | 0.664 | 3.321 | 92.191 |  |  |  |  |
| 10 | 0.429 | 2.147 | 94.338 |  |  |  |  |
| 11 | 0.367 | 1.834 | 96.172 |  |  |  |  |
| 12 | 0.252 | 1.261 | 97.434 |  |  |  |  |
| 13 | 0.194 | 0.969 | 98.402 |  |  |  |  |
| 14 | 0.117 | 0.586 | 98.989 |  |  |  |  |
| 15 | 0.080 | 0.399 | 99.387 |  |  |  |  |
| 16 | 0.060 | 0.299 | 99.687 |  |  |  |  |
| 17 | 0.032 | 0.161 | 99.848 |  |  |  |  |
| 18 | 0.021 | 0.103 | 99.951 |  |  |  |  |
| 19 | 0.006 | 0.031 | 99.982 |  |  |  |  |
| 20 | 0.004 | 0.018 | 100.000 |  |  |  |  |

**TABLE S4.** Components and coefficient sets used in the analysis

| Cytokines | Components | | | | | |
| --- | --- | --- | --- | --- | --- | --- |
|  | 1 | 2 | 3 | 4 | 5 | 6 |
| EGF | 0.055 | 0.227 | -0.259 | 0.605 | 0.165 | -0.193 |
| GM-CSF | 0.666 | 0.539 | 0.094 | -0.265 | -0.220 | 0.060 |
| IFN-γ | 0.808 | 0.134 | -0.065 | 0.176 | 0.090 | -0.367 |
| IL-1β | 0.583 | -0.773 | -0.016 | 0.106 | 0.047 | -0.028 |
| IL-4 | 0.572 | -0.130 | -0.134 | -0.306 | 0.403 | 0.086 |
| IL-5 | 0.833 | -0.468 | -0.048 | 0.034 | 0.184 | 0.106 |
| IL-6 | 0.733 | -0.612 | -0.007 | 0.094 | 0.063 | -0.046 |
| IL-8 | 0.335 | 0.352 | -0.128 | 0.718 | 0.114 | 0.101 |
| IL-9 | 0.546 | 0.530 | -0.098 | -0.281 | 0.152 | -0.250 |
| IL-10 | 0.486 | 0.350 | -0.034 | -0.032 | 0.309 | 0.586 |
| IL-13 | 0.789 | -0.092 | 0.164 | -0.052 | -0.319 | 0.051 |
| IL-17 | 0.317 | 0.397 | -0.015 | -0.430 | 0.354 | -0.013 |
| IL-23 | 0.733 | -0.636 | 0.006 | 0.090 | 0.014 | -0.015 |
| IL-33 | -0.088 | -0.020 | 0.938 | 0.008 | 0.314 | -0.044 |
| Leptin | 0.246 | -0.035 | 0.229 | 0.081 | -0.478 | 0.525 |
| sRAGE | 0.076 | 0.185 | -0.307 | 0.014 | 0.560 | 0.215 |
| TGF-β1 | 0.730 | 0.462 | 0.147 | -0.099 | -0.350 | -0.093 |
| TNF-α | 0.866 | 0.317 | 0.091 | -0.038 | -0.179 | -0.149 |
| TSLP | -0.088 | -0.051 | 0.927 | 0.021 | 0.301 | -0.074 |
| VEGF | 0.216 | 0.434 | 0.296 | 0.665 | 0.026 | 0.066 |
